# Identification of the miRNA signature and key genes in colorectal cancer lymph node metastasis

**DOI:** 10.1186/s12935-021-02058-9

**Published:** 2021-07-07

**Authors:** Xi Wang, Guangyu Gao, Zhengrong Chen, Zhihao Chen, Mingxiao Han, Xiaolu Xie, Qiyuan Jin, Hong Du, Zhifei Cao, Haifang Zhang

**Affiliations:** 1 Department of Clinical Laboratory, The Second Affiliated Hospital of Soochow University, No. 1055 San Xiang Road, Suzhou, 215004 Jiangsu China; 2 Department of Oncology, The Second Affiliated Hospital of Soochow University, Suzhou, People’s Republic of China; 3 Department of Gastrointestinal Surgery, The Second Affiliated Hospital of Soochow University, Suzhou, People’s Republic of China; 4 Department of Orthopedics, The Second Affiliated Hospital of Soochow University, Suzhou, People’s Republic of China; 5 Department of Pathology, The Second Affiliated Hospital of Soochow University, No. 1055 San Xiang Road, Suzhou, 215004 Jiangsu China

**Keywords:** MicroRNA, Colorectal cancer, Lymph node metastasis, Prognostic signature, HS3ST2

## Abstract

**Background:**

Because its metastasis to the lymph nodes are closely related to poor prognosis, miRNAs and mRNAs can serve as biomarkers for the diagnosis, prognosis, and therapy of colorectal cancer (CRC). This study aimed to identify novel gene signatures in the lymph node metastasis of CRC.

**Methods:**

GSE56350, GSE70574, and GSE95109 datasets were downloaded from the Gene Expression Omnibus (GEO) database, while data from 569 colorectal cancer cases were also downloaded from The Cancer Genome Atlas (TCGA) database. Differentially expressed miRNAs (DE-miRNAs) were calculated using R programming language (Version 3.6.3), while gene ontology and enrichment analysis of target mRNAs were performed using FunRich (http://www.funrich.org). Furthermore, the mRNA–miRNA network was constructed using Cytoscape software (Version 3.8.0). Gene expression levels were verified using the GEO datasets. Similarly, quantitative real-time PCR (qPCR) was used to examine expression profiles from 20 paired non-metastatic and metastatic lymph node tissue samples obtained from patients with CRC.

**Results:**

In total, five DE-miRNAs were selected, and 34 mRNAs were identified after filtering the results. Moreover, two key miRNAs (hsa-miR-99a, hsa-miR-100) and one gene (heparan sulfate-glucosamine 3-sulfotransferase 2 [*HS3ST2*]) were identified. The GEO datasets analysis and qPCR results showed that the expression of key miRNA and genes were consistent with that obtained from the bioinformatic analysis. A novel miRNA–mRNA network capable of predicting the prognosis and confirmed experimentally, hsa-miR-99a-HS3ST2-hsa-miR-100, was found after expression analysis in metastasized lymph node tissue from CRC samples.

**Conclusion:**

In summary, miRNAs and genes with potential as biomarkers were found and a novel miRNA–mRNA network was established for CRC lymph node metastasis by systematic bioinformatic analysis and experimental validation. This network may be used as a potential biomarker in the development of lymph node metastatic CRC.

**Supplementary Information:**

The online version contains supplementary material available at 10.1186/s12935-021-02058-9.

## Background

Colorectal cancer (CRC) is a serious health threat worldwide. Compared to the early stage of disease, the treatment response and overall survival of patients with advanced CRC is still very poor. The 5-year survival rate of patients with advanced CRC is reduced from 50 to 10% [1]. Surgical tumor resectioning is still the cornerstone of the treatment of localized, advanced CRC. There is no cure for metastatic tumors that cannot be surgically removed, or that respond poorly to the effects of chemotherapy and radiotherapy [2]. At present, the AJCC’s TNM staging system has limited value in predicting recurrence [3, 4]. Moreover, while lymph node metastasis is not the only form of metastasis in patients with advanced CRC, it is one of the most essential prognostic risk factors [5]. So as to promote the prognosis and individualized treatment, it is therefore urgent to determine the key factors influencing lymph node metastasis in CRC.

MicroRNAs (miRNAs) are small noncoding RNAs, associated with post-transcriptional gene regulation [6]. According to previous studies, miRNAs can regulate many target genes, or many genes can regulate one type of miRNA [7]. Notably, Sin et al. found that some miRNAs can improve the therapeutic effect by improving the drug sensitivity of cancer cells [8]. Ma et al. found that miR-374a, miR-92a, and miR-106a increased drug resistance and promoted growth and metastasis of lung cancer [9]. Similarly, Kania et al. reported that miR-9-3p and miR-9-5p decreased DNA topoisomerase IIα expression levels in acquired resistance to etoposide and may act as a biomarker of responsiveness to TOP2-targeted therapy [10]. However, the mechanisms of miRNAs in the transformation of adenomas to adenocarcinoma remain unknown.

In recent years, an accumulating number of studies have documented that bioinformatics analyses have provided a deeper understanding of the aberrant genetic pathways in the development, progression, and metastasis of various human cancers. Amongst others, these included investigations into breast [11], lung [12], liver [13], and colorectal cancer [14]. There are many reports describing the identification of key genes or pathways in CRC or for predicting CRC prognosis by integrated bioinformatics analysis [15–18]. Although Zhang et al. identified key candidate genes and constructed novel miRNA–mRNA regulatory axes in CRC liver metastasis, and found that miR-885 promoted CRC cell migration by decreasing the expression of von Willebrand factor (vWF) and insulin-like growth factor binding protein 5 (IGFBP5) by using integrated bioinformatics analysis and in vitro experiments [19], studies identifying biomarkers associated with CRC metastasis by bioinformatics analysis are scare. In particular, the systematic analyses of mRNAs and miRNAs in CRC lymph node metastasis is still not adequate enough to definitively determine aberrant genetic pathways. In this study, a systematic bioinformatics analysis identified two miRNAs (hsa-miR-99a and hsa-miR-100) and one gene (heparan sulfate-glucosamine 3-sulfotransferase 2 [HS3ST2]), through which a novel mRNA–miRNA regulatory network in lymph node metastasis of CRC was established. This model may be used in the early diagnosis and therapy of metastatic CRC.

## Materials and methods

### Microarray data

The Gene Expression Omnibus (GEO) database (https://www.ncbi.nlm.nih.gov/geo) is a public, functional genomics data repository that allows users to download and import gene expression data. In this study, gene expression data were obtained from GEO for GSE56350, GSE70574, and GSE95109.

GSE56350 includes data from eight primary CRC tissue types derived from stage II–III CRC patients with (n = 20) or without (n = 15) lymph node metastasis. MicroRNA expression profiling analysis of these samples was performed using an Agilent-021827 Human miRNA Microarray assay [miRNA_107_Sep09_2_105]. GSE70574included data from 16 T1-stage CRCs comprised of seven lymph node-positive and nine lymph node-negative tumors that were processed using an Agilent-031181 Unrestricted_Human_miRNA_V16.0_Microarray 030840 assay (Feature Number version). GSE95109 contained data from 13 lymph node-negative patients and nine lymph node-positive patients. The mRNA profiles of all 22 patients were analyzed using Agilent microarray technology to explore the differential expression between lymph node-negative and lymph node-positive subgroups.

### Differentially expressed miRNAs and mRNAs analysis

R programming language (Version 3.6.3) was used to compare the two groups of tissue. Cut-off criteria were established by |log_2_FC|≥ 1, while P < 0.05 indicated significant statistical differences [20].

### Functional and pathway enrichment analyses

In this study, transcription factor enrichment analysis was conducted and transcription factors that may regulate differentially expressed miRNAs **(**DE-miRNAs) were identified by FunRich (http://www.funrich.org) which is a publicly accessible software with the ability to identify enriched transcription factors. While, the target genes of the DE-miRNAs were also analyzed by Gene Ontology (GO) and Kyoto Encyclopedia of Genes and Genomes (KEGG) enrichment analysis through the DIANA-miRPath v3.0 database (http://www.microrna.gr/miRPathv3/).

### Construction of PPI network and clustered subnetworks

The exploration of protein interactions helps to reveal the underlying pathological mechanism of CRC. In this study, the Search Tool for the Retrieval of Interacting Genes/Proteins (STRING) database (https://string-db.org/) was used to construct a protein–protein interaction network.

### Prediction of miRNA target genes and the miRNA–mRNA regulatory network

A previous study suggested that the function of miRNAs lies in the regulation of target genes. Therefore, the prediction of target genes is particularly important as it can indirectly elucidate the biological function and enrichment pathway of the associated miRNAs. The miRNA enrichment function in FunRich was used for miRNA targeting predictions. By combining the FunRich and differential analysis results from the GSE95109 data, screened genes were identified, and the miRNA–mRNA regulatory network built using Cytoscape software (Version 3.8.0).

### Construction of a prognostic signature model

To determine the influence of differential expression of miRNAs on the prognosis of CRC patients, univariate and multivariate Cox proportional risk regression analysis was performed for different miRNA expression levels. Those miRNAs found to be related to CRC prognosis were selected, and a linear risk model established based on The Cancer Genome Atlas (TCGA) dataset. Collectively, data from 569 CRC patients downloaded from TCGA were randomly divided into training and test groups. A model capable of predicting the genetic features of input data was built into the training group, with validity testing being performed using the test group data. First, the training group was analyzed by univariate Cox regression analysis to select the prognoses-related differentially expressed genes (DEGs). Through further functional analysis and development of potential risk characteristics, the least absolute shrinkage and selection operator (LASSO) method was used to regress the high-dimensional prediction factors as reported previously [21, 22]. The R “glmnet” package was used to calculate the coefficient and partial likelihood deviation [23]. Through multivariate Cox regression analysis, the identified miRNAs were further studied to determine significant targets and build a linear risk model. To better understand the relationship between the selected miRNAs and the prognosis of CRC patients, a risk prediction model was constructed. By using the “survival ROC” package in R programming language (Version 3.6.3), the AUCs of the ROC curves associated with 3 and 5 year survival were constructed to assess the predictive power of the identified miRNAs, respectively.

### Hematoxylin and eosin (H&E) staining and analysis

Fresh colorectal carcinoma and lymph node tissue samples were fixed in 10% formalin and embedded in paraffin before sectioning and staining. Tissue sections that were 4 μm thick were respectively deparaffinized and rehydrated in a series of xylene and ethanol steps. H&E staining was performed according to standard protocols.

### Verification of miRNA and mRNA expression

Twenty non-metastatic, and 20 metastatic lymph node CRC tissue samples were obtained from patients with CRC, respectively. Ethical approval was obtained from the Second Affiliated Hospital of Soochow University ethics committee. Total RNA was extracted from the tissue samples using TRIzol reagent (Mesgen Biotech Co., Shanghai, China), and treated with RNase-free DNase I (Takara Biotech Co., Dalian, China) to eliminate traces of mixed DNA. Then, PCR was employed to confirm that there was no genomic DNA contamination using the specific primers of target genes. The reverse transcription performed according to the manufacturer’s protocol. Real-time PCR was performed on the QuantStudio 5 Real-Time PCR System (Thermo Fisher Scientific, Shanghai, China) using qPCR SYBR Green master mix (Vazyme Biotech Co., Nanjing, China). The primers are shown in Additional file 2: Table S1. The expression levels of the miRNAs were normalized against U6 (internal standard control) and calculated using the 2^−ΔΔCt^ method. All experiments were performed in triplicate.

### Statistical analysis

Negative and positive lymph node tests were performed to evaluate the statistical significance between the two groups. All data analysis was performed using R programming language (version 3.6.3) and GraphPadPrism6 (GraphPad Software, Inc., La Jolla, California, USA). The correlation between the miRNAs and their possible targeted mRNA among individual samples were also assessed. P < 0.05 was viewed as indicating statistical significance. Using the CORR function, the correlation analysis between the RT-qPCR and RNA-seq results was calculated in Excel 2013 (Microsoft Corporation, Redmond, WA, USA).

## Results

### Identification of the miRNAs between non-metastatic and metastatic lymph node tissues

**Fig. 1:**
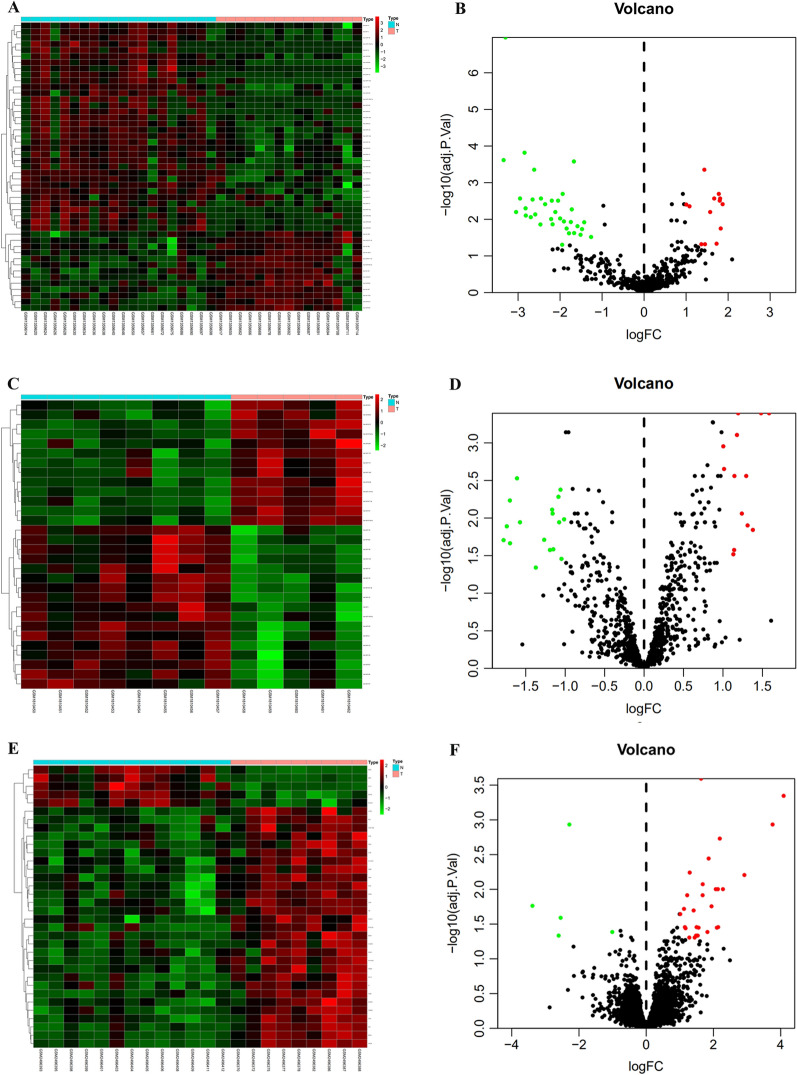
Volcano and heat maps of GSE56350, GSE70754 and GSE95109. **A** Unsupervised clustering analysis of differentially-expressed miRNAs (DE-miRNAs) in GSE56350. **B** Volcano plots of miRNAs in GSE56350. **C** Unsupervised clustering analysis of DE-miRNAs in GSE70754. **D** Volcano plots of miRNAs in GSE70754. **E** Unsupervised clustering analysis of the DE-mRNAs in GSE95109. **F** Volcano plots of mRNAs in GSE95109. **A**, **C**, **E** Red dots indicate significantly up-regulated miRNAs or mRNAs, green dots indicate significantly down-regulated miRNAs or mRNAs. **B**, **D**, **F** Red dots indicate up-regulated DE-miRNAs or DE-mRNAs, green dots indicate down-regulated DE-miRNAs or DE-mRNAs, black dots indicate non-differentially expressed miRNAs or mRNAs

**Fig. 2:**
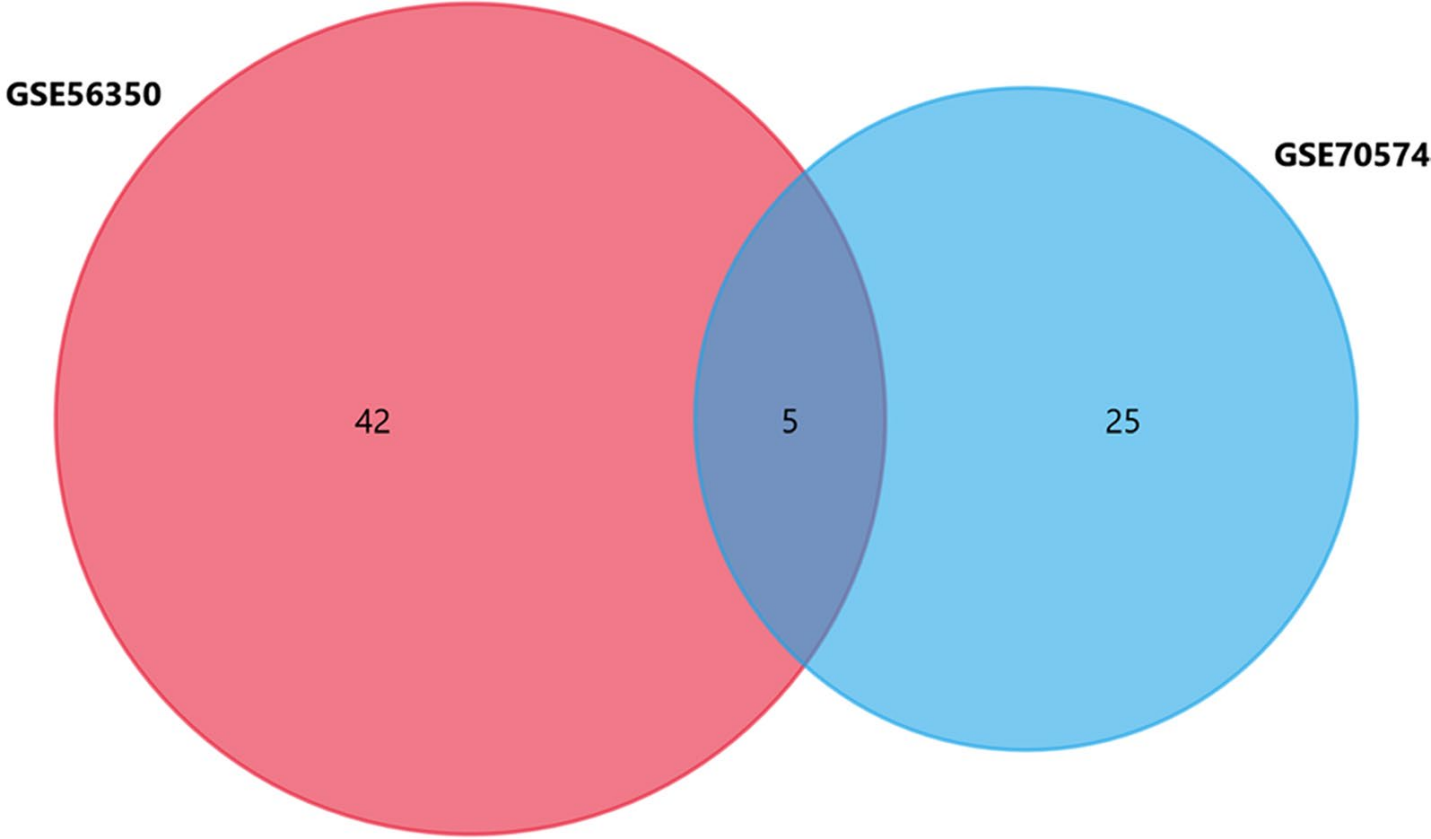
Venn diagram of GSE56350 and GSE70574

We performed a comprehensive bioinformatics analysis to elucidate the key miRNA–mRNA axis in the metastatic lymph node of CRC (flow chart was shown in Additional file 1: Figure S1). Using R programming language (Version 3.6.3) to research the gene expression profiles, a total of three GEO datasets (GSE56350, GSE70574 and GSE95109) were selected and downloaded. In total, 47 DE-miRNAs (13 upregulated and 34 downregulated), and 30 DE-miRNAs (13 upregulated and 17 downregulated) were obtained from the GSE56350 and GSE70574 datasets, respectively. Similarly, 34 DEGs (29 upregulated and 5 downregulated, Additional file 3: Table S2) were identified from the GSE95109 dataset according to the cut-off criteria for this study (P < 0.05 and |log2FC|≥ 1). The identified DE-miRNAs and DEGs are shown in heat maps as well as a volcano plots (Fig. 1A–F). Furthermore, a total of five common DE-miRNAs were screened from the GSE56350 and GSE70574 datasets. This included five downregulated miRNAs (Fig. 2), the details of which are listed in Additional file 4: Table S3.

### Screening of potential transcription factors and enrichment analysis

**Fig. 3:**
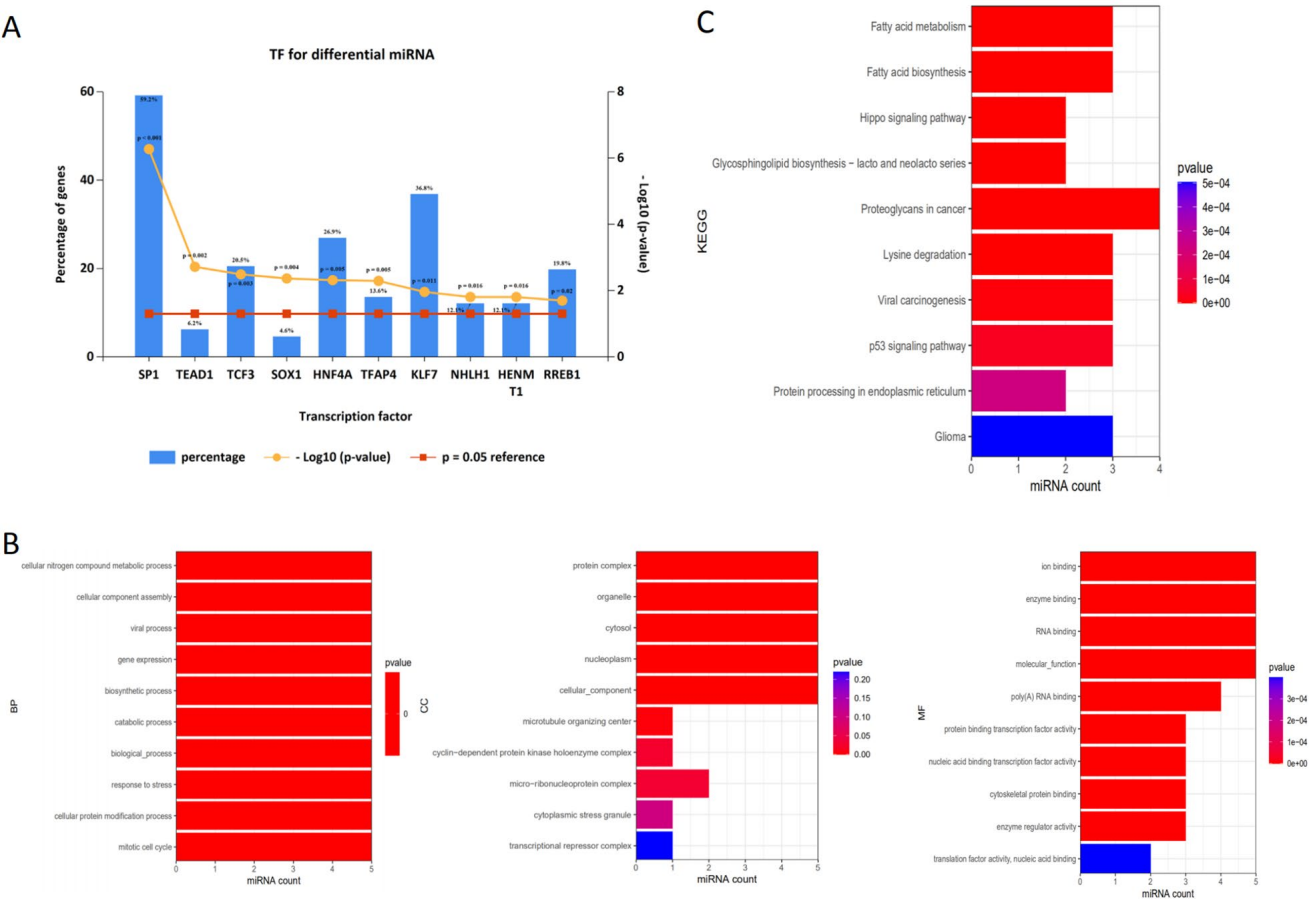
Screening of potential transcription factors and target genes of DE-miRNAs. **A** Identification of the potential transcription factors of DE-miRNAs by FunRich software. **B** The top 10 of biological process, cellular component, and molecular function of the target genes of DE-miRNAs. **C** KEGG pathway enriched by potential target mRNAs of DE-miRNAs

**Fig. 4:**
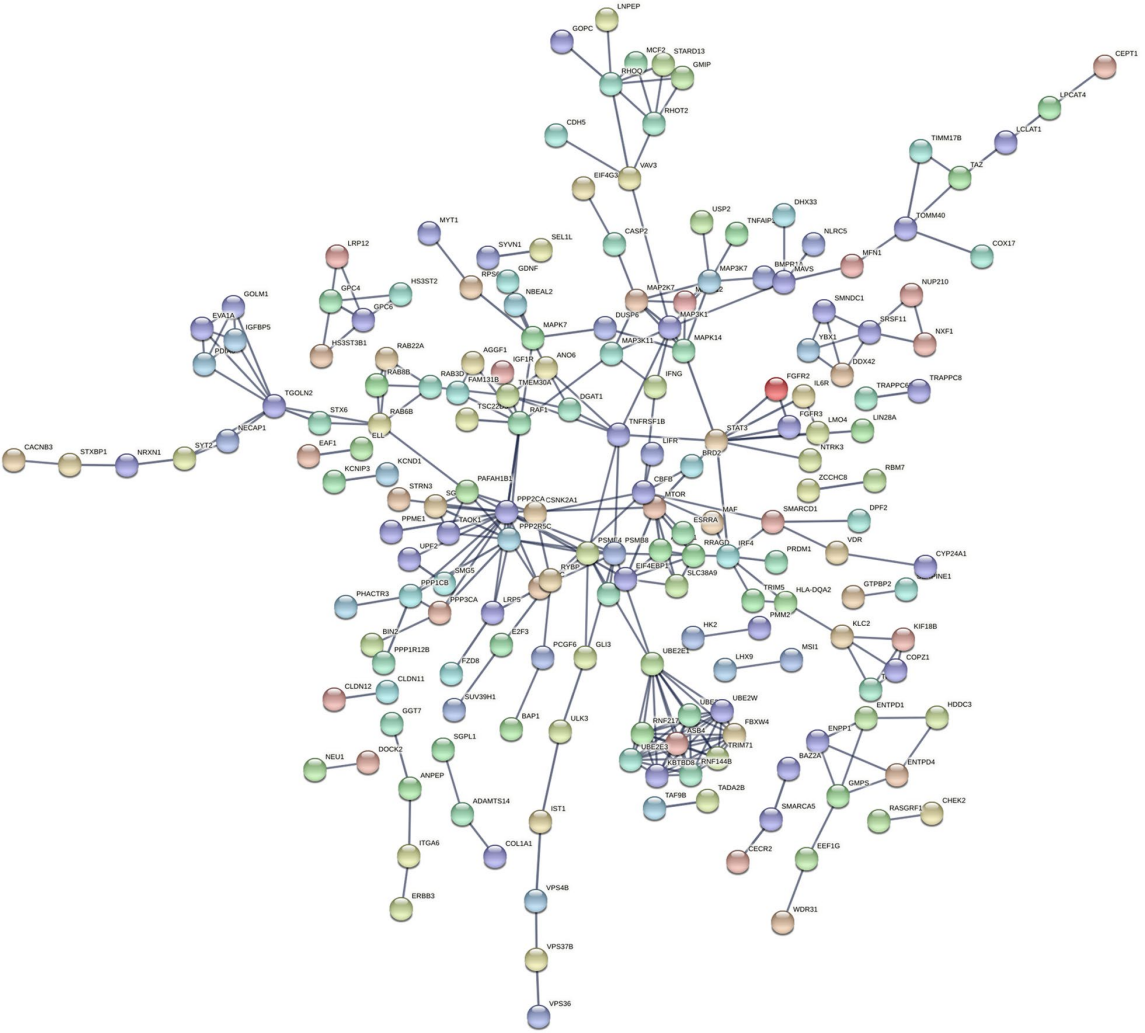
The PPI network of the target genes of the identified DE-miRNAs

To identify the shared transcriptional factor signatures associated with the DE-miRNAs, FunRich was used. As shown in Fig. 3A, the top 10 transcription factors that were found to be enriched for included SP1, TEAD1, TCF3, SOX1, HNF4A, TFAP4, KLF7, NHLH1, HENMT1, and RREB1. The results of GO analysis for the target genes of DE-miRNAs showed that DE-miRNAs were most enriched in the cellular nitrogen compound metabolic process, protein complex, ion binding, etc. (Fig. 3B). In addition, these target genes were mainly enriched in the pathways including fatty acid metabolism, fatty acid biosynthesis, hippo signaling pathway, proteoglycans in cancer, lysine degradation and so on by KEGG pathway analysis (Fig. 3C). The construction of the protein–protein interaction (PPI) network is shown in Fig. 4. Under the conditions that the comprehensive Gt score > 0.7 and unconnected points were removed, this network included 598 nodes and 1004 edges.

### Construction of miRNA–mRNA regulatory network

**Fig. 5:**
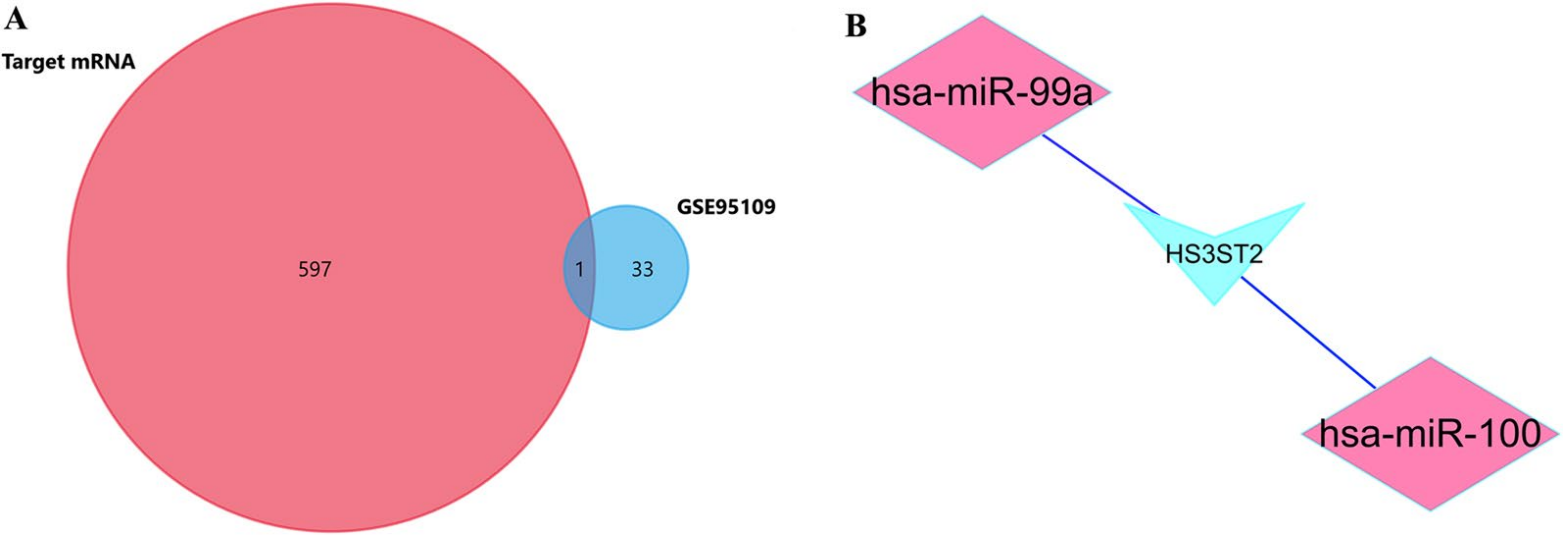
The miRNA–mRNA network of CRC lymph node metastasis. **A** Venn diagram of target mRNAs of DE-miRNAsand DE-mRNAs of GSE95109. **B** The miRNA–mRNA regulatory network in the lymph node metastasis of CRC

The 598 potential target genes of screened DE-miRNAs were predicted using FunRich. The subsequent Venn diagram analysis of the target mRNA and GSE 95109-derived DEGs identified one gene of interest (Fig. 5A). To show the composition and relationship between target genes more intuitively, a complete network of target genes was constructed using Cytoscape (Version 3.8.0). Finally, two essential miRNA–mRNA regulatory networks that demonstrated the crucial effects of lymph node metastasis in CRC were identified (Fig. 5B).

### Construction of a prognostic risk model and predictability assessment

**Fig. 6:**
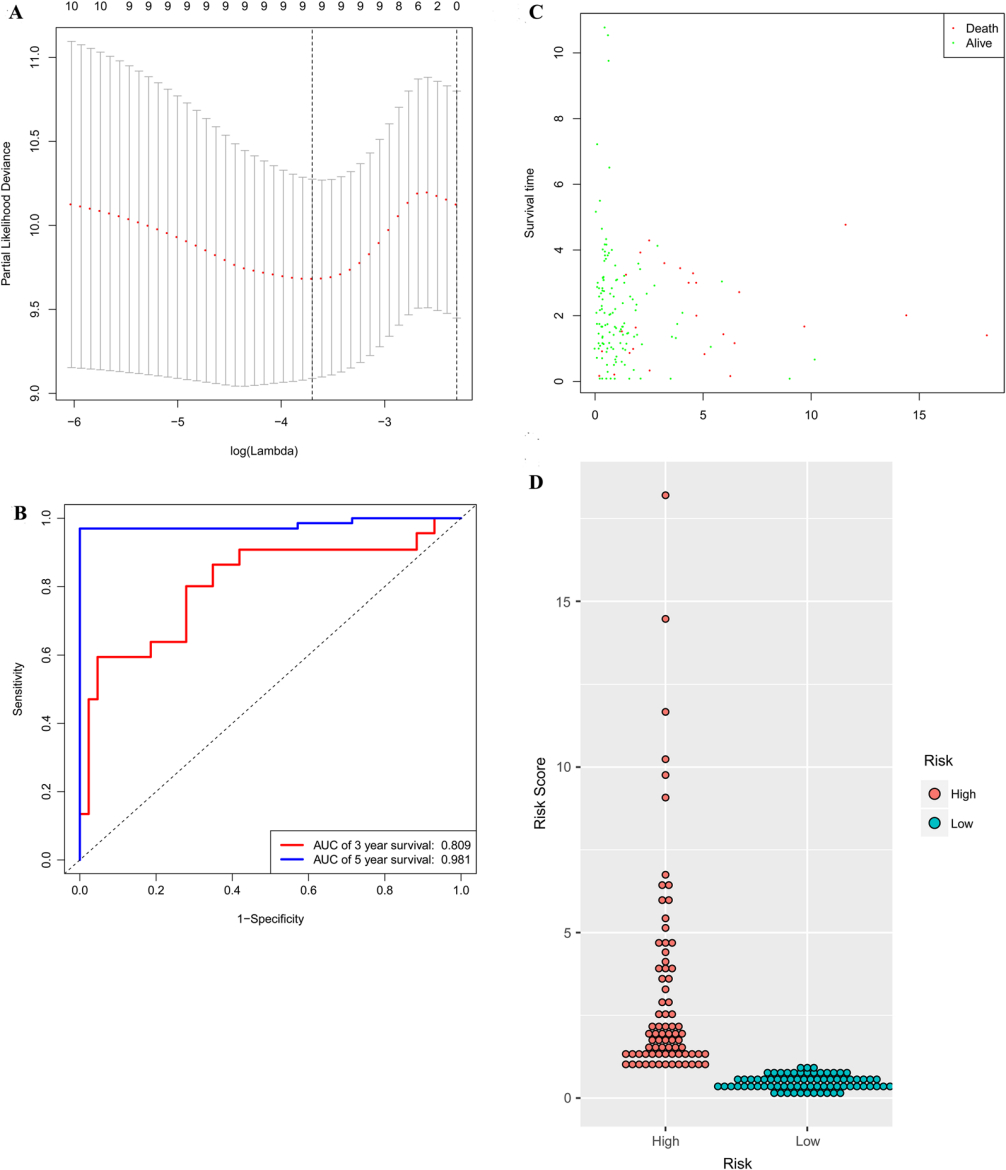
Identification of the signature significantly associated with the survival of patients with CRC in the training group. **A** LASSO Cox regression algorithm was used to reduce the scope. **B** Time-dependent ROC curves analysis. **C**, **D** Risk score distribution and survival status for patients in high- and low-risk groups by the signature. *LASSO* least absolute shrinkage and selection operator, *ROC* receiver operating characteristic

To identify the best prognostic miRNAs, the LASSO Cox regression algorithm was applied to 20 prognosis-related miRNAs. Nine miRNAs were selected to build the risk signature based on the minimum standard (Fig. 6A). Multivariate Cox proportional risk regression analysis was subsequently conducted using the nine candidate prognostic miRNAs to assess their independent prognostic values. According to the Cox model, seven candidate miRNAs (hsa-miR-125a-5p, hsa-miR-377, hsa-miR-100, hsa-miR-455-3p, hsa-miR-126, hsa-miR-199a, and hsa-miR-99a) showed significance and were selected as independent prognostic factors. These seven prognostic miRNAs were then combined to build a model to predict patient outcomes. The AUC for 3 year survival using the 7-miRNA signature achieved a value of 0.809, while the 5 year survival AUC value was calculated as 0.981. This demonstrated that the model performed well in predicting the survival risk of CRC patients (Fig. 6B). According to this risk model, patients were divided into high and low risk groups. The results showed that this model accurately predicted the clinical outcomes of patients. The risk scores, survival status and distribution of the expression levels of the seven miRNAs in each patient were also analyzed (Fig. 6C, D).

### Validation of the expression of DE-miRNAs and DEGs

**Fig. 7:**
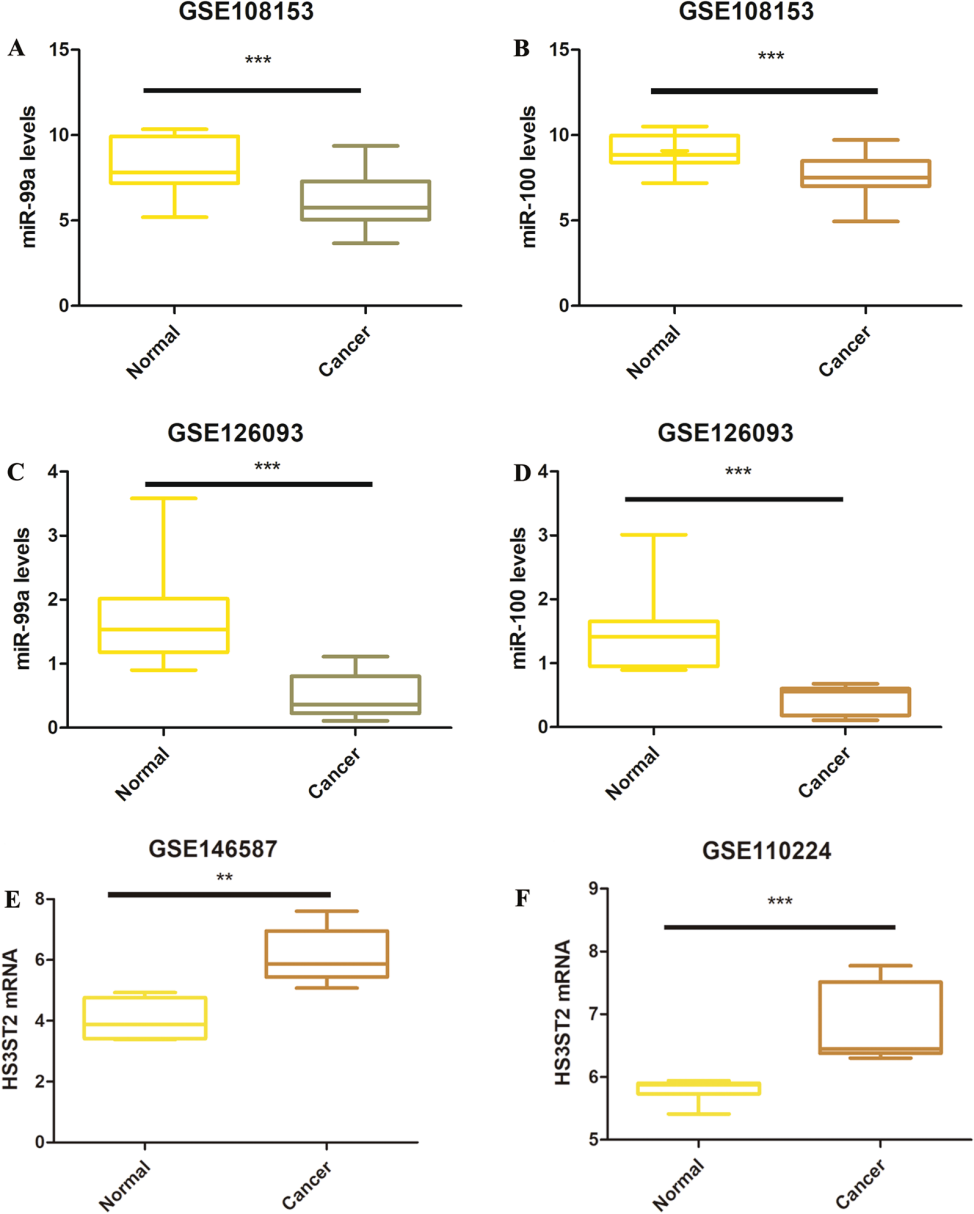
The differential expression of hsa-miR-99a and hsa-miR-100 in CRC tissues and their corresponding normal-appearing tissues. **A**–**D** Validation of hsa-miR-99a and hsa-miR-100 in GEO datasets GSE108153 and GSE126093, respectively. **E**, **F** Validation of HS3ST2 in GEO datasets GSE146587 and GSE110224, respectively. **P < 0.01, ***P < 0.001

**Fig. 8:**
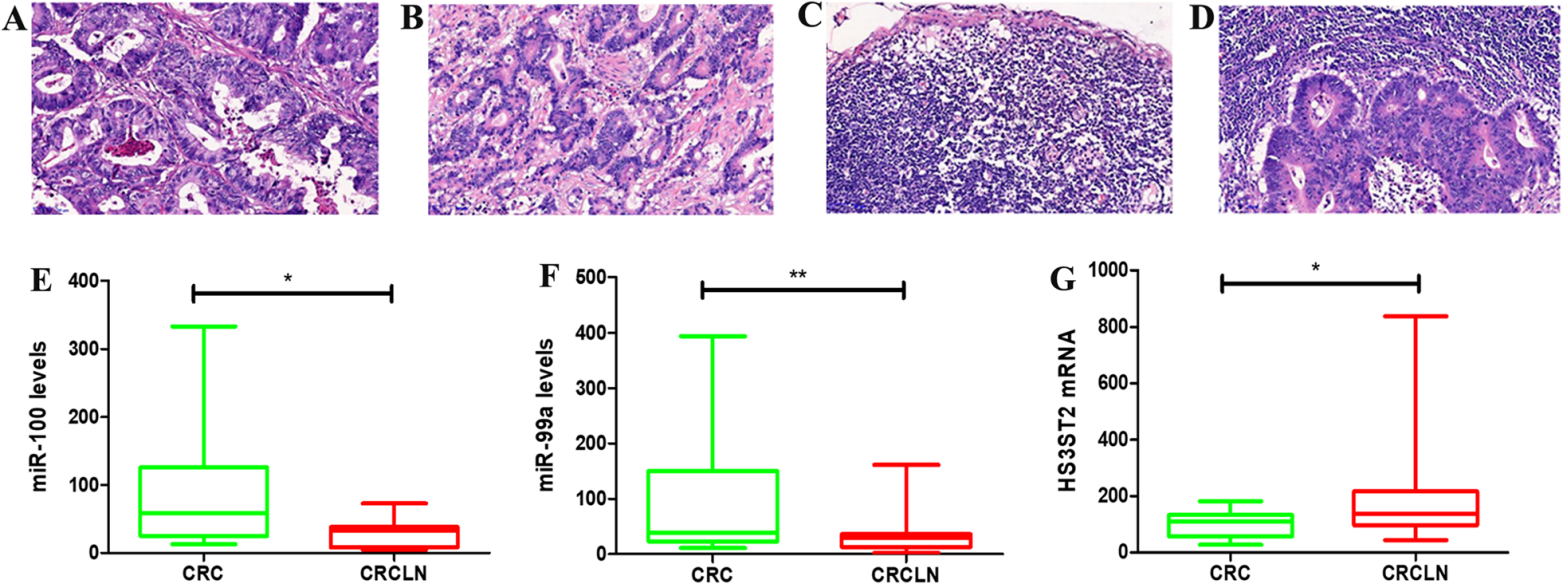
The expression levels of hsa-miR-100, hsa-miR-99a and HS3ST2 in CRC tissues and lymph node metastatic CRC tissues. **A** Hematoxylin and eosin (H&E) staining of CRC tissues without lymph node metastasis. **B** H&E staining of CRC tissues in patients with lymph node metastatic CRC. **C** H&E staining of lymph nodes in patients with CRC. **D** H&E staining of metastatic lymph nodes in patients with CRC. **E**–**G** Validation of hsa-miR-99a, hsa-miR-100 and HS3ST2 in GEO datasets GSE146587 and GSE110224, respectively. *P < 0.05, **P < 0.01

In order to further identify the key genes in CRC lymph node metastasis, the expression of hsa-miR-99a, hsa-miR-100, and *HS3ST2* were assessed using the GEO database. Two different GEO datasets (GSE108153 and GSE126093) showed that hsa-miR-99a and hsa-miR-100 were down-regulated in CRC tissue compared to normal tissue (Fig. 7A–D, P < 0.001), while data from GSE146587 and GSE110224 demonstrated that *HS3ST2* was up-regulated in CRC tissue compared to the normal tissue(Fig. 7E, P < 0.001; F, P < 0.01). Moreover, the expression of hsa-miR-99a, hsa-miR-100, and HS3ST2 was validated by qPCR using 20 CRC tissue samples with lymph node metastasis and 20 CRC tissue samples without lymph node metastasis (Fig. 8A–D). As shown in Fig. 8E–G, the expression of hsa-miR-99a and hsa-miR-100 decreased significantly in CRC tissue with lymph node metastasis compared to CRC tissue without lymph node metastasis (P < 0.01, P < 0.05), while the expression of *HS3ST2* greatly increased in CRC tissue with lymph node metastasis (P < 0.05). Generally, the data indicated that hsa-miR-100, hsa-miR-99a, and *HS3ST2* could be the candidate biomarkers for CRC lymph node metastasis.

## Discussion

CRC can occur through the progression of adenomas, which is the result of genetic and epigenetic events in epithelial cells. Some microarray-based studies have identified gene expression profiles in adenomas and cancers [24–26]. In this study, five DE-miRNAs were identified, and it suggested that these five DE-miRNAs played a role in promoting the development of CRC. Functional annotation indicated that these five DE-miRNAs were mostly related to the cellular nitrogen compound metabolic process, protein complex and RNA binding. This is consistent with the recognition that the cellular nitrogen compound metabolic process and protein complex play important roles in tumor development [27, 28]. KEGG pathway analysis indicated that the target genes of five DE-miRNAs were mainly enriched in ten pathways, including fatty acid metabolism, fatty acid biosynthesis, hippo signaling pathway, proteoglycans in cancer, lysine degradation and so on. It was reported that the dysregulation of fatty acids synthesis/catabolism played a regulatory role in the metabolic regulation that supports cancer cell growth [29, 30]. Hippo signaling pathway is a key regulator of organ size, tissue hemostasis and regeneration. Dysregulation of the Hippo pathway has been recognized in a variety of human cancers, including pancreatic cancer [31]. Proteoglycans are dominant components of the extracellular matrix, which are not only a highly heterogeneous proteome, but also an attractive pharmacological target in cancer [32]. However, these pathways have not been reported in the pathogenesis of CRC or in the lymph node metastasis of CRC yet.

A miRNA–mRNA regulatory network was built following FunRich and Cytoscape software analysis. Five DE-miRNAs (hsa-miR-100, hsa-miR-375, hsa-miR-125b, hsa-miR-143 and hsa-miR-99a) and one potential DEG were identified by combining two screening results. It has previously been reported that down-regulation of hsa-miR-100 is involved in tumorigenesis and progression of multiple cancer types [33] In addition, low expression of miR-99a significantly predicts poor prognosis in head and neck squamous cell carcinoma and regulates cancer cell migration and invasion [34]. Xu et al. also demonstrated that miR-99a inhibited the migratory and invasive abilities of cancer cells by regulating the expression of the insulin-like growth factor 1 receptor. It has therefore been concluded that the miR-99a/IGF1R axis may provide novel insight into the pathogenesis of gastric cancer [35]. Hsa-miR-125b, as a tumor suppressor, can contribute to prostate tumorigenesis by modulations in the PI3K/AKT and MAPK/ERK signaling pathways. These key pathways simultaneously influence prostate cancer progression [36]. Hsa-miR-125b can also inhibit the development of bladder cancer by inhibiting SIRT7 and MALAT1 [37], and has additionally been found to play an essential role in the progression of oral squamous cell cancer (OSCC), as well as the target genes and transcription factors associated with hsa-miR-125b [38]. In this study, survival analysis indicated that overexpression of hsa-miR-125b was related to worse overall survival in patients with CRC by using KM-plot software.

Through the combination of the GEO and TCGA analysis, two miRNAs (hsa-miR-100 and hsa-miR-99a) were especially notable. We found that hsa-miR-100 and hsa-miR-99a had different expression levels in non-metastasized and metastasized lymph node tissue through qPCR analysis. Interestingly, hsa-miR-100 and hsa-miR-99a both targeted HS3ST2. HS3ST2, an enzyme mediating 3-*O*-sulfation of heparan sulfate, is present in all cell types and tissues. It interacts with growth factors, tyrosine kinase receptors, matrix metalloproteinases and extracellular matrix proteins to modulate cell adhesion, proliferation and motility [39, 40]. In breast, colorectal, lung, cervical, pancreatic and recurrent prostate cancers, HS3ST2 is silenced due to hypermethylation, suggesting that it may play an important role in multiple cancers [41]. A previous study has confirmed that abnormal HS3ST2 methylation levels were important in endometrial hyperplasia and carcinogenesis [42]. The prognostic significance of HS3ST2 mRNA expression in several cancer types has been evaluated [43, 44]. HS3ST2 protein expression could be used as a favorable prognostic tissue biomarker in patients with primary advanced-stage lung cancer [44]. For gastric cancer, statistical analyses using a Chi-squared test showed that there is a significant difference in HS3ST2 methylation levels between gastric cancer and non-cancerous patients. It has therefore been concluded that HS3ST2 methylation may act as a novel cancer-related molecular mechanism for the detection of new treatment strategies [45]. In this study, we found that HS3ST2 was upregulated in the metastatic lymph node CRC tissue compared to CRC samples. Moreover, a novel miRNA–mRNA network, hsa-miR-99a-HS3ST2-hsa-miR-100, has been described for the first time in the metastasized lymph node samples derived from CRC.

Our findings proved that many differentially expressed mRNAs and miRNAs were involved in the lymph node metastasis of CRC by select signaling pathways, and that they have prognostic significance. Because most of our data were generated by applying bioinformatics tools to data from the GEO database and TCGA platform, and given that a limited number of relevant samples were available for analysis, more data analysis and clinical experiments should be performed to further develop these potential biomarkers for predicting the recurrence of CRC.

Taken together, this study revealed potential mechanisms associated with the development of CRC. Several differentially expressed mRNAs and miRNAs were identified across non-metastasized and metastasized lymph node tissues by using bioinformatics methods. Hsa-miR-100, hsa-miR-99a and HS3ST2 were determined to be potential biomarkers for predicting the recurrence of CRC.

## Supplementary Information

**Additional file 1: Figure S1.** A whole flow chart of this study.

**Additional file 2: Table S1.** The primers for verification of miRNA expression and mRNA expression.

**Additional file 3: Table S2.** Identification of DEGs associated with lymph node metastasis of CRC

**Additional file 4: Table S3.** Identification of DE-miRNAs associated with lymph node metastasis of CRC

## Data Availability

The datasets used and/or analyzed during the current study are available from the corresponding author on reasonable request.

## Abbreviations

GEO: Gene Expression Omnibus
KEGG: Kyoto Encyclopedia of Genes and Genomes
miRNA: MicroRNAs
TCGA: The Cancer Genome Atlas
ROC: Receiver operating characteristic
AUC: Area under the curve
qPCR: Quantitative real-time PCR
DE-miRNAs: Differentially expressed miRNAs
DEGs: Differentially expressed genes
CRC: Colorectal cancer
HS3ST2: Heparan sulfate-glucosamine 3-sulfotransferase 2
