# Supplementary figures and images for: Identification of the miRNA signature and key genes in colorectal cancer lymph node metastasis
Source: Cancer Cell Int. 2021 Jul 7;21:358. doi: 10.1186/s12935-021-02058-9 (PMC8314594; doi:10.1186/s12935-021-02058-9)

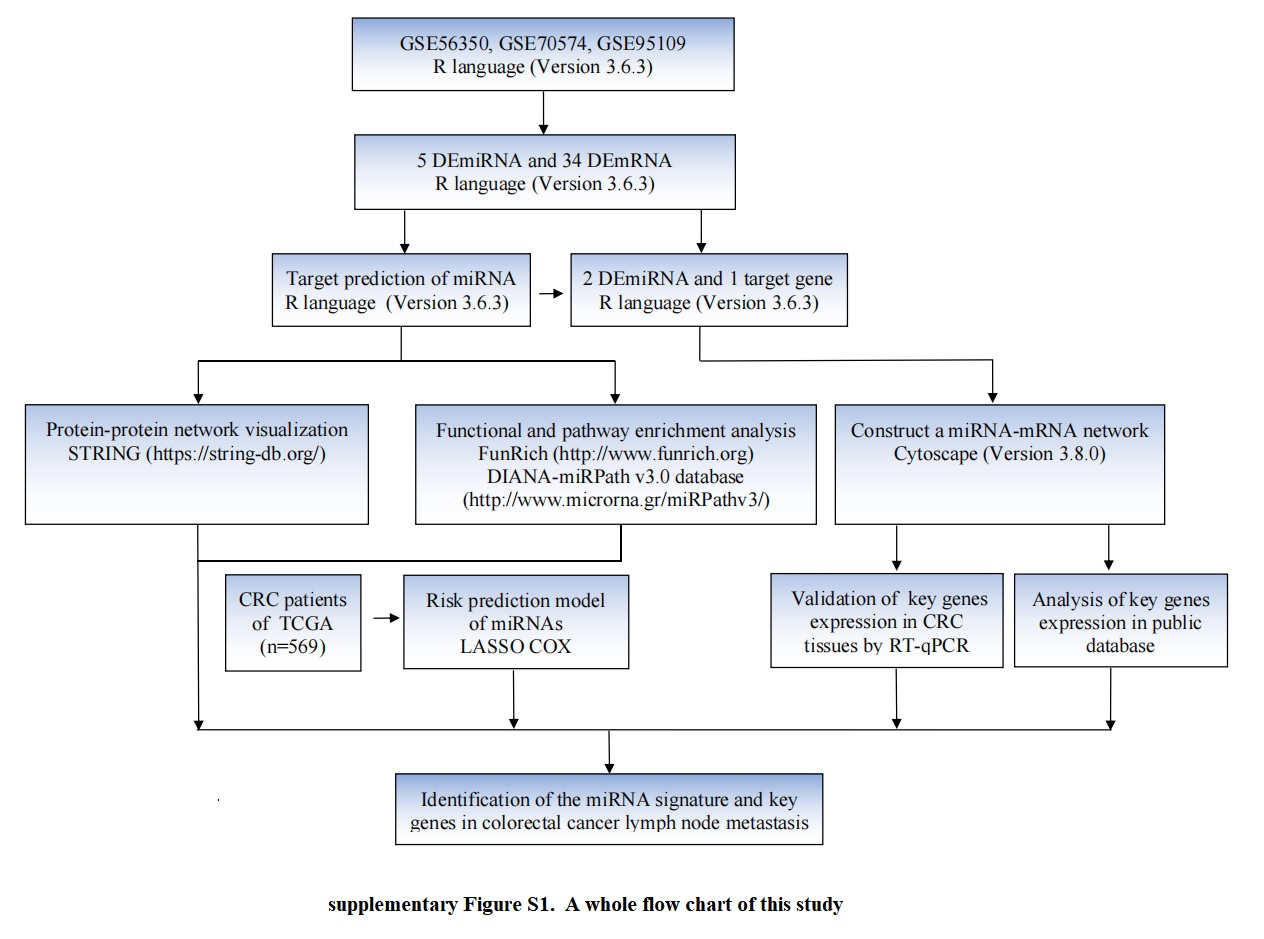

Supplement: Supplementary file 1 — Additional file 1: Figure S1. A whole flow chart of this study. [file 12935_2021_2058_MOESM1_ESM.jpg]
