# Supplementary material for: Identification of the miRNA signature and key genes in colorectal cancer lymph node metastasis
Source: Cancer Cell Int. 2021 Jul 7;21:358. doi: 10.1186/s12935-021-02058-9 (PMC8314594; doi:10.1186/s12935-021-02058-9)
Supplement: Supplementary file 2 — Additional file 2: Table S1. The primers for verification of miRNA expression and mRNA expression. [file 12935_2021_2058_MOESM2_ESM.docx]

| **Primer sets name** | **Reverse transcriptase primers (5' to 3')** | **RT-qPCR primers (5' to 3')** |
| --- | --- | --- |
| has-mir-100 | GTCGTATCCAGTGCAGGGTCCGAGGTATTCGCACTGGATACGACCACAAGT | F: CAGTGCAGGGTCCGAGGTAT R: CGCGAACCCGTAGATCCGAA |
| has-mir-99a | GTCGTATCCAGTGCAGGGTCCGAGGTATTCGCACTGGATACGACCACAAGA | F: CAGTGCAGGGTCCGAGGTAT R: CGTATAACCCGTAGATCCGAT |
| U6 | GTCGTATCCAGTGCAGGGTCCGAGGTATTCGCACTGGATACGACAAAATA | F: AGAGAAGATTAGCATGGCCCCTG R: ATCCAGTGCAGGGTCCGAGG |
| HS3ST |  | F: GGCTGGATTGGTACAGGAGC R: CCACGATCAGCTTGGTGTCT |
| β-actin |  | F: CTCCATCCTGGCCTCGCTGT R: GCTGTCACCTTCACCGTTCC |

**Table S1. The primers for verification of miRNA expression and mRNA expression**
