# Supplementary material for: Identification of the miRNA signature and key genes in colorectal cancer lymph node metastasis
Source: Cancer Cell Int. 2021 Jul 7;21:358. doi: 10.1186/s12935-021-02058-9 (PMC8314594; doi:10.1186/s12935-021-02058-9)
Supplement: Supplementary file 3 — Additional file 3: Table S2. Identification of DEGs associated with lymph node metastasis of CRC [file 12935_2021_2058_MOESM3_ESM.doc]

**Table S2. Identification of DEGs associated with lymph node metastasis of CRC**

| **ID** | **logFC** | **AveExpr** | **t** | **P.Value** | **adj.P.Val** |
| --- | --- | --- | --- | --- | --- |
| MMP3 | -3.383380607 | 2.743291273 | 8.441456328 | 1.62E-08 | 0.000255365 |
| MMP1 | -2.604327371 | 3.872452102 | 7.849649847 | 5.74E-08 | 0.000451091 |
| CCL11 | -2.545017495 | 4.141617407 | 7.216135246 | 2.33E-07 | 0.001165698 |
| WNT5A | -2.285951543 | 6.469308505 | -7.110177522 | 2.97E-07 | 0.001165698 |
| PCDH18 | -1.008646403 | 3.110155251 | 6.808152934 | 5.92E-07 | 0.001861911 |
| SLAMF6 | 1.012364852 | 3.136288745 | 6.447210686 | 1.37E-06 | 0.00359875 |
| HLA-DOA | 1.124220113 | 3.019812643 | 6.185459043 | 2.55E-06 | 0.00573321 |
| PVRIG | 1.150652615 | 4.417614191 | 6.095269942 | 3.17E-06 | 0.006222161 |
| TRIM22 | 1.169702283 | 4.947332919 | 5.9191503 | 4.83E-06 | 0.008443899 |
| CCR7 | 1.219016804 | 2.731750916 | 5.803821202 | 6.39E-06 | 0.00994535 |
| CD3E | 1.281657782 | 5.313206737 | 5.75769866 | 7.14E-06 | 0.00994535 |
| VPREB3 | 1.293250555 | 3.746465497 | 5.732721287 | 7.59E-06 | 0.00994535 |
| CCDC102B | 1.409808703 | 4.144533696 | 5.589740914 | 1.08E-05 | 0.012154586 |
| LTB | 1.440290668 | 6.533277848 | 5.587143365 | 1.08E-05 | 0.012154586 |
| SASH3 | 1.480906594 | 4.121667145 | -5.415593938 | 1.65E-05 | 0.017272876 |
| C16orf54 | 1.498350606 | 5.088107616 | 5.3829589 | 1.79E-05 | 0.017546428 |
| LILRA4 | 1.503138459 | 4.132907382 | 5.323556627 | 2.07E-05 | 0.019115674 |
| CD69 | 1.531925947 | 3.32492286 | 5.279049319 | 2.31E-05 | 0.020148371 |
| C7 | 1.556824402 | 4.306873776 | 5.191476469 | 2.86E-05 | 0.022719827 |
| MS4A1 | 1.632143731 | 4.377301176 | -5.119251869 | 3.43E-05 | 0.025641473 |
| CD52 | 1.678266234 | 2.85855908 | 4.96443047 | 5.03E-05 | 0.035009824 |
| CD22 | 1.681388143 | 3.364439797 | 4.941125861 | 5.33E-05 | 0.035009824 |
| PCOLCE2 | 1.821419427 | 6.325012753 | 4.940084735 | 5.34E-05 | 0.035009824 |
| HLA-DOB | 1.856948346 | 3.984899196 | 4.907577183 | 5.80E-05 | 0.035619973 |
| IGHM | 1.942704339 | 3.267700289 | 4.893964811 | 5.99E-05 | 0.035619973 |
| SELL | 2.068994543 | 4.119056992 | 4.86316037 | 6.47E-05 | 0.03634284 |
| TRAT1 | 2.101207727 | 4.919100006 | -4.765556311 | 8.26E-05 | 0.041118009 |
| BANK1 | 2.131469636 | 3.586958156 | 4.747757343 | 8.63E-05 | 0.041118009 |
| GZMK | 2.140117625 | 3.461884833 | 4.67721575 | 0.00010293 | 0.046348051 |
| FCMR | 2.187861765 | 4.307929701 | -4.675107745 | 0.000103474 | 0.046348051 |
| HS3ST2 | 2.281337525 | 5.157835286 | 4.656340675 | 0.000108439 | 0.046348051 |
| CCL19 | 2.919633409 | 3.986878293 | 4.642180581 | 0.000112343 | 0.046477613 |
| CR2 | 3.758336449 | 7.863578434 | 4.561609892 | 0.000137398 | 0.049439631 |
| FDCSP | 4.088409432 | 5.323765484 | 4.560403115 | 0.000137813 | 0.049439631 |
