# Supplementary material for: Identification of the miRNA signature and key genes in colorectal cancer lymph node metastasis
Source: Cancer Cell Int. 2021 Jul 7;21:358. doi: 10.1186/s12935-021-02058-9 (PMC8314594; doi:10.1186/s12935-021-02058-9)
Supplement: Supplementary file 4 — Additional file 4: Table S3. Identification of DE-miRNAs associated with lymph node metastasis of CRC [file 12935_2021_2058_MOESM4_ESM.doc]

**Table S3. Identification of DE-miRNAs associated with lymph node metastasis of CRC**

| **ID** | **logFC** | **AveExpr** | **t** | **P.Value** | **adj.P.Val** |
| --- | --- | --- | --- | --- | --- |
| hsa-miR-99a | -3.2998 | 5.878924 | -9.07224 | 1.47E-10 | 1.08E-07 |
| hsa-miR-100 | -2.35603 | 6.687555 | -4.37844 | 0.00011 | 0.003868 |
| hsa-miR-125b | -2.95247 | 5.767905 | -4.71345 | 4.12E-05 | 0.002706 |
| hsa-miR-143 | -3.34466 | 5.496491 | -5.96943 | 9.83E-07 | 0.000242 |
| hsa-miR-375 | -1.95009 | 7.292945 | -3.1063 | 0.003834 | 0.049641 |
